# Collecting quantitative experimental data from a non-WEIRD population: challenges and practical recommendations from a field experiment in rural Sierra Leone

**DOI:** 10.1186/s13104-021-05828-w

**Published:** 2021-11-17

**Authors:** Hanna Luetke Lanfer, Doreen Reifegerste, Sorie Ibrahim Kargbo

**Affiliations:** 1 School of Public Health, Bielefeld University, Universitätsstraße 25, 33615 Bielefeld, Germany; 2 Freetown, Sierra Leone

**Keywords:** Survey, Methodology, WEIRD, Cross-cultural, Sierra Leone

## Abstract

**Objective:**

Standardized pretest–posttest experimental designs with quantitative surveys are frequently applied to evaluate the effectiveness of health programs. However, this method is strongly informed by research on samples from Western, Educated, Industralized, Rich, and Democratic (WEIRD) societies and may not produce meaningful results in a distinct cultural, educational and socioeconomic context.

**Results:**

This paper reports several methodological challenges encountered along the research process of collecting quantitative survey data (i.e., during recruitment, obtaining informed consent, matching pretest–posttest data and data collection) for a mixed-methods field experiment on domestic handwashing in Sierra Leone. Ethical dilemmas of certain research practices are pointed out and potential solutions or alternatives are recommended for each challenge. Analysis of these challenges highlights the importance of reflecting on the aptness of research methodologies for non-WEIRD samples. While this is not to say that quantitative surveys are not suitable in a non-WEIRD context, their employment require considerable time for extensive pilot testing, involving local interviewers and participants in designing research projects and the modification of data collection strategies.

**Supplementary Information:**

The online version contains supplementary material available at 10.1186/s13104-021-05828-w.

## Introduction

Public health professionals are required to produce evidence of the effectiveness of programs to inform evidence-based practice [1, 2]. In this, standardized pretest–posttest experimental designs with quantitative surveys to measure changes in knowledge, attitudes or self-reported health behavior before and after interventions are frequently applied for data collection [3]. Scholars have pointed out that while these are prevalent research designs and frequently used methods, they are strongly informed by research on samples from Western, Educated, Industralized, Rich, and Democratic (WEIRD, 4) societies. Applying the same methods to non-WEIRD populations might not produce meaningful results and ignore the cultural, educational and socioeconomic context of participants [5, 6]. The present study describes challenges encountered during a mixed-methods field experiment aiming to increase domestic handwashing, which was carried out by researchers originating from a WEIRD (Germany) and non-WEIRD society (Sierra Leone) and implemented in a non-WEIRD country (Sierra Leone). Further details about the study design can be found in [7, 8]. This study was planned and implemented under the lead of the first author in collaboration with a local investigator, the third author. In addition, two senior researchers, conducting clinical studies in Sierra Leone, revised our research plan prior to data collection. Research participants were not involved in designing the research project. Despite careful considerations and pilot testing prior to the investigation, various methodological challenges in recruitment, matching of pretest–posttest data and data collection arose during the study. It is our hope that reflecting on our experiences and sharing recommendations will increase discussions about methodologies for non-WEIRD participants.

## Main text

### Setting and study design

The present study was carried out to test the effectiveness of two different intervention strategies with the objective to increase domestic handwashing in a sample of four rural villages in Bombali District in northern Sierra Leone (see [7] for the research design and the survey in Additional file 1) with *n* = 233 mostly illiterate, low-income participants (Table 1).

**Table 1:** Demographic characteristics of survey participants

Characteristic	Total		Group 1	Group 2	Group 3	Group 4
	*n*	%	*n*	*n*	*n*	*n*
Participants per group						
Pretest	240	100.0	60	60	60	60
Posttest	233	97.1	57	59	60	57
Gender^a^						
Male	117	50.2	29	29	30	29
Female	116	49.8	28	30	30	28
Estimated age						
18–25	34	14.6	6	12	7	9
26–35	53	22.7	15	10	15	13
36–45	63	27.0	11	19	19	14
46–59	57	24.5	17	12	15	13
60+	17	7.6	6	4	3	4
Missing	9	3.9	2	2	1	4
Occupation						
Farmer	183	78.5	50	48	41	44
Business man/woman	19	8.2	2	2	10	5
Student	13	5.6	2	6	3	2
Teacher	7	3.0	1	0	3	3
Other	11	4.7	2	3	3	3
Education						
No formal education	188	80.7	50	45	54	38
Primary school	33	14.2	4	10	3	16
Secondary school	7	3.0	1	2	1	3
College	5	2.1	2	1	2	0
Religion						
Muslim	164	70.4	44	28	43	49
Christian	69	29.6	13	31	17	8

^a^All characteristics, except ‘participants per group’ refer to data collected during the posttest

The field experiment was implemented between February and October 2019 (Table 2).

**Table 2:** Timeline of the field experiment

Month	Activity
February 2019	Recruitment of four villages; 60 survey participants in each village
March 2019	Pretest survey and observation wave
April–August 2019	Monthly (i.e. five) interventions according to treatment conditions, each followed by a focus group discussion
September 2019	Posttest survey and observation wave
October 2019	Evaluation meetings in all villages to collect feedback and share preliminary results

While data was collected via different qualitative and quantitative instruments (i.e., surveys, observations and focus group discussions), the quantitative survey was most problematic and we, thus, focus on the challenges encountered with the survey. The survey was translated from English to Krio, following the procedures suggested by Tsang et al. [9]. These included forward and backward translation, pilot testing with potential participants and construct and content validation before data collection. Due to high illiteracy rates in the analyzed sample, survey data was collected in face-to-face interviews by the first and third author. Interviews lasted between 15 and 50 min. Participants received a local meal after each of the monthly interventions as compensation.

### Methodological challenges

The methodological challenges described in this section are aligned with the procedures of the research process, i.e., starting with recruitment to data collection.

#### Recruiting diverse participants

Access to the ‘field’ was only possible via the local chief and other community elders who act as gatekeepers. While gatekeepers during recruitment procedures are not an unknown phenomenon, especially in organizational contexts, authors [10] have pointed out that there is little guidance on how to engage with gatekeepers in non-Western or non-democratic contexts to access marginalized or less powerful citizens for study participation. Furthermore, in a study examining the power local chiefs in Sierra Leone exercise in different social realms [11], it was shown that chiefs prioritized their own families to participate in activities associated with access to social, financial and other resources. To avoid a bias towards the powerful, a detailed list of criteria was given to the chief, indicating for instance that no more than two people per household could participate and calling for equal shares of men and women of different ages. Visits to participants’ homes suggested that we had indeed captured participants from different families.

An additional barrier to recruitment were language barriers among women. Sierra Leone is a multi-linguistic country with at least 18 different languages, yet despite its linguistic diversity, the only official languages of the country are English and Krio [12]. As Krio is the dominant local language throughout the country [13] and has a written and oral expression, it was chosen for data collection. In Bombali District, where fieldwork took place, two other tribes and their languages (Temne and Limba) prevail. While recruiting a sufficient number of Krio-speaking men was not a challenge, women were less likely to be bilingual. To include an equal number of female participants, women with good Krio listening comprehension but limited speaking skills were exclusively interviewed by the third author who is fluent in several local languages. While the survey questions were asked in Krio for all participants, some females responded in their preferred language and answers were translated and entered into the software accordingly by the third author.

#### Obtaining informed consent

Informed consent was obtained from all survey participants as a part of the standard ethical procedures. This practice originates from changes in Western medical practice to increase patient’s autonomy and self-determination [14]. Despite its widespread acceptance, it has been described as incongruent with the cultural and social values of rural, low literate, collectivistic groups as found in sub-Saharan Africa [15, 16]. To comply with ethical requirements and be sensitive to the cultural meaning of signing forms with unknown content in a setting in which individual autonomy is embedded in the wider community, all 60 participants were gathered in a communal place. The consent form in written English was handed out to any literate community member present while it was orally translated to the preferred local language (Limba, Krio or Temne). The literate community members verified it was congruent with the written form. Participants had the opportunity to ask the researchers questions and discuss participation among themselves. Thumbprinting as a form of signing for the illiterate was done in the presence of an impartial witness.

#### Matching pretest–posttest data

Matching data from the same respondent at two or more points of data collection while maintaining participant’s anonymity is a quality indicator of panel studies [17]. Therefore, participants are generally asked to generate a unique, non-identifiable code based on a systematic pattern, e.g., with permanent data such as birthdate, birth location, phone number and own name [17, 18]. However, these data are not applicable to match the responses of illiterate participants in rural Sierra Leone. First, participants had little recorded data about themselves, e.g., few people knew their birthdate, there are no precise addresses in a village and individual phone ownership is low. Second, identical names are common as first names are generally given to honor a person, for instance, children are often named after their parents. Further, there is not much variety with regards to family names either as large, often polygamous families are found in this setting. Last, other sociodemographic data (e.g., tribe, religion, occupation) was the same for most participants. For a gap in the academic literature on how to address this challenge, we attributed people numerical codes, printed on colored cardboard to be brought back for the posttest survey. In addition, each name and assigned code were recorded offline in a notebook to comply with data protection regulations. After 6 months, 28 out of 291 people had lost their cards and 76 obviously had the wrong code, mismatching their sociodemographic data from the first survey. This might be explained by the fact, that some participants were observed showing their codes to each other and picked up the wrong card afterwards due to being unable to distinguish them. Using demographic data, we could allocate 59 cases, but 15.5% (*n* = 45) remained unallocated. To create more memorable, self-generated codes, we propose the use of different simple visual cards that participants pick themselves, e.g., a certain symbol or a series of different symbols [19].

#### Likert scales

**Fig. 1:**
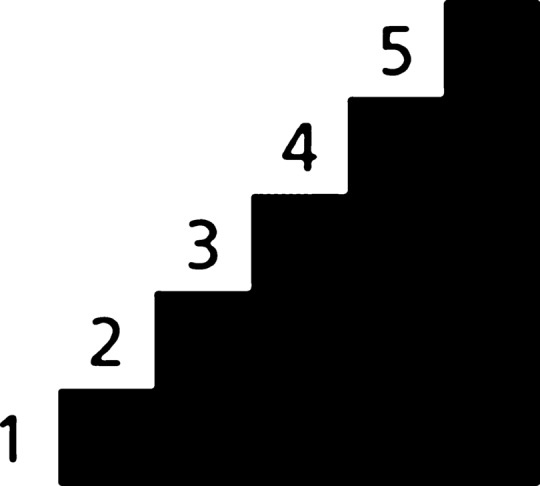
Depiction of the visual analog scale

About half of the survey questions were presented as Likert scales, the most frequent instrument to measure psychometric variables in WEIRD samples [4]. With slight adaptations, e.g., using a visual analog instead of a numerical scale, they are also applied in non-WEIRD populations [20]. While we tested two visual scales and opted for a scale in the form of stairs which improved the quality of answers with our pretest sample (see Fig. 1), the literature is scarce on other challenges with psychometric questions.

In Sierra Leone, however, scale-rated, psychometric questions proved to be difficult for the majority of our participants who either tended to stop using the scale after a few of the actual questions, saying only “yes” or “no”, solely pointed to the extreme ends of the scale, alternated between the values four and five or appeared to point randomly at different values while giving an oral explanation that contradicted their choice. Items with a negation appeared to be most confusing and numerous participants asked the interviewers for help to answer them. These response patterns may be due to a high proportion of illiterate participants and unfamiliarity with scale-rated questions. Moreover, the participants’ collective orientation should also be considered in relation to two observed response patterns in collective-oriented groups [21, 22], known as the Extreme Response Style (i.e., a tendency to only use the endpoints of the answer scale [23]), and Acquiescence Response Style (i.e., a tendency to agreeing with the questions despite other options [24]). Apart from psychometric, close-ended questions, the survey included open-ended questions (e.g., What is the difference between using soap for handwashing or just water without soap?) and answers were classified to certain predefined responses by the interviewer. While it took considerably longer to collect data from open-ended questions as the respondents tended to give lengthy answers, they might be more preferable in such a context as participants appeared more at ease and the data provided meaningful insights. Moreover, as research concepts are largely unknown, reassuring participants that the survey was not an exam and making a joke also appeared to be a useful strategy with participants who became nervous during the interview.

#### Numerical questions

Asking for people’s precise age and other numerical questions (e.g., In how many interventions did you participate?) are widely used questions in survey research, assuming at least basic numerical skills among the participants. Given the low educational level and lack of personal data in our sample, questions requiring a numerical response were problematic. While some participants could estimate their approximate age and were consequently allocated to five age groups, other responses given were obviously wrong, e.g., when a white-haired, older woman said she was 12 years old. In these cases, the researchers with the help of the chief allocated the participant to an age group. Moreover, several questions requiring numerical responses were excluded from the analysis as the results were not reliable. A method using local beads to indicate quantities that was employed among low literate women in Uganda [25] is worth exploring with participants of similar educational backgrounds.

### Discussion

Our reflections highlight some of the challenges and limitations we encountered when collecting survey data with participants of a non-WEIRD population in Sierra Leone. Despite an awareness of the local context, collaborations with local investigators in the planning process, pilot testing and numerous adaptations, we were faced with several issues, ranging from recruitment to conducting the survey interview itself. We also pointed out ethical dilemmas of certain research practices, e.g., obtaining written informed consent from illiterate participants from a collective-oriented society.

While this is not to say that quantitative surveys are not suitable for non-WEIRD populations, there should be more reflections about alternative methods of data collection and necessary adaptations to meet the needs of the participants and produce meaningful data. Based on our experiences, we have made several practical recommendations to manage these challenges which should also be included in guidelines of ethical commissions. Moreover, we suggest planning for a considerable amount of time to be spent on additional and prolonged pilot testing with iterative adaptions, involving local interviewers and participants in designing research projects, and to stay flexible for data collection strategies.

## Limitations

This study was carried out in Sierra Leone and our reflections provide insights into the challenges encountered in a specific setting. Our findings and recommendations might not be applicable to a different non-WEIRD population or a more heterogenous setting, i.e., in a multicultural group. However, it is hoped that scholars embarking on studies in a comparable context will be better informed and equipped to deal with challenges to be met during fieldwork in a non-WEIRD context.

## Supplementary Information

**Additional file 1. **Pretest–posttest survey on hand hygiene (field experiment).

## Data Availability

The datasets used and/or analyzed during the current study are available from the corresponding author on reasonable request.

## Abbreviation

WEIRD: Western, Educated, Industrialized, Rich, and Democratic

## References

[1] Rossmann C, Holtzhausen DR, Zerfass A (2014). Strategic health communication: theory- and evidence-based campaign development. The Routledge handbook of strategic communication.

[2] Windsor R (2015). Evaluation of health promotion and disease prevention programs.

[3] Friemel TN, Frey T, Rossmann C, Hastall MR (2019). Kommunikationskampagnen zur Gesundheitsförderung und Prävention. Handbuch der Gesundheitskommunikation.

[4] Henrich J, Heine SJ, Norenzayan A (2010). The weirdest people in the world?. Behav Brain Sci.

[5] Batres C, Borras-Guevara ML, Perrett DI (2018). Collecting data cross-culturally: methodological challenges that arise when testing non-WEIRD populations.

[6] Tuhiwai SL (2012). Decolonizing methodologies: research and indigenous peoples.

[7] Luetke Lanfer H (2021). Through a lens of scarcity: health communication in a low-income context.

[8] Luetke Lanfer H, Reifegerste D (2021). Embracing challenging complexity: exploring handwashing behavior from a combined socioecological and intersectional perspective in Sierra Leone. BMC Public Health.

[9] Tsang S, Royse CF, Terkawi AS (2017). Guidelines for developing, translating, and validating a questionnaire in perioperative and pain medicine. Saudi J Anaesth.

[10] Kalina M, Scott D (2019). You have to say everything is nice here. QRJ.

[11] Acemoglu D, Reed T, Robinson JA (2014). Chiefs: economic development and elite control of civil society in Sierra Leone. J Polit Econ.

[12] Statistics Sierra Leone (2016). 2015 Population and housing census: summary of final results.

[13] Gellman M (2020). Mother tongue won’t help you eat: language politics in Sierra Leone. Afr J Pol Sci Int Relat.

[14] Frimpong-Mansoh A (2008). Culture and voluntary informed consent in African health care systems. Dev World Bioeth.

[15] Afolabi MO, Okebe JU, McGrath N, Larson HJ, Bojang K, Chandramohan D (2014). Informed consent comprehension in African research settings. Trop Med Int Health.

[16] Appiah R (2021). Gurus and Griots: revisiting the research informed consent process in rural African contexts. BMC Med Ethics.

[17] Kristjansson AL, Sigfusdottir ID, Sigfusson J, Allegrante JP (2014). Self-generated identification codes in longitudinal prevention research with adolescents: a pilot study of matched and unmatched subjects. Prev Sci.

[18] Ripper L, Ciaravino S, Jones K, Jaime MCD, Miller E (2017). Use of a respondent-generated personal code for matching anonymous adolescent surveys in longitudinal studies. J Adolesc Health.

[19] Luetke Lanfer H, Rossmann C. Challenges to generate unique, anonymous participant codes for a longitudinal study among illiterate participants in rural Sierra Leone. In: WAPOR Conference, October 6–8, Salamanca, Spain. 2020.

[20] Kopper S, Parry K. Introduction to measurement and indicators. Research Resources. Abdul Latif Jameel Poverty Action Lab. 2021. https://www.povertyactionlab.org/resource/introduction-measurement-and-indicators.

[21] Bachman JG, O'Malley PM, Freedman-Doan P. Response styles revisited: racial/ethnic and gender differences in extreme responding. Monitoring the Future Occasional Paper 2010;72:1–18. https://deepblue.lib.umich.edu/bitstream/handle/2027.42/137850/occ72.pdf?sequence=1&isAllowed=y.

[22] Benítez I, He J, van de Vijver FJR, Padilla J-L (2016). Linking extreme response style to response processes: a cross-cultural mixed methods approach. Int J Psychol.

[23] Hui CH, Triandis HC (1989). Effects of culture and response format on extreme response style. J Cross Cult Psychol.

[24] Marin G, Gamba RJ, Marin BV (1992). Extreme response style and acquiescence among hispanics. J Cross Cult Psychol.

[25] Bwambale FM, Moyer CA, Komakech I, Mangen F-W, Lori JR (2013). The ten beads method: a novel way to collect quantitative data in rural Uganda. J Public Health Res.

