# Supplementary material for: Collecting quantitative experimental data from a non-WEIRD population: challenges and practical recommendations from a field experiment in rural Sierra Leone
Source: BMC Res Notes. 2021 Nov 17;14:414. doi: 10.1186/s13104-021-05828-w (PMC8597296; doi:10.1186/s13104-021-05828-w)
Supplement: Supplementary file 1 — Additional file 1. Pretest–posttest survey on hand hygiene (field experiment). [file 13104_2021_5828_MOESM1_ESM.docx]

**Pretest-posttest survey on hand hygiene (field experiment)**

**Introduction**

Good morning/afternoon. My name is ___________________ We are conducting a survey on hand washing and I would like to interview you at two different points in time.

[Explain consent form and rights of the participant in detail.]

Each survey takes about 15 – 20 minutes to complete.

At this time, do you want to ask me anything about this survey?

Should I proceed with the questions?

| **No** | | **Variable** | | **Question text** | | **Items** | | **Answer options** | | **Information for interviewer** | | |
| --- | --- | --- | --- | --- | --- | --- | --- | --- | --- | --- | --- | --- |
| 1 | | Intervention group | | *Filled in by interviewer* | |  | | - IG 1 - IG 2 - IG 3 - IG 4 | | One choice, assign code to participant | | |
| 2 | | Sex | | *Filled in by interviewer* | |  | | - Male - Female | | One choice | | |
| 3 | | Age | | How old are you? | |  | | - 18-25 - 26-35 - 36-45 - 46-59 - 60+ - No answer | | One number | | |
| 4 | | Marital status | | What is your legal status? | |  | | - Single - Married (legally and/or traditionally) - Separated/divorced - Widowed - No answer | | One choice | | |
| 5a | | Children | | Do you have children? | |  | | - Yes - No - No answer | | One choice | | |
| 5b | | Children | | How many children under the age of five years do you have? | |  | | *Fill in exact number* | | Open question, one number | | |
| 6 | | Household | | If living with family, how many people share your household right now? | |  | | *Fill in exact number* | | Open question, one number | | |
| 7 | | Education | | What is your highest level of education? | |  | | - No formal schooling - Primary school - Secondary school - College - University - No answer | | One choice | | |
| 8 | | Work, profession | | What do you do for a living? | |  | | - Teacher - Farmer - Businessman/ woman - Housewife/ husband - Nurse/medical worker/CHW - Chief - Contractor/ handyman - Bike rider - Other - No answer | | One choice | | |
| 9 | | Social life, position in community | | What is your role in this community? | |  | | - Ordinary community member - Chief - Elder - Traditional Healer - Religious leaders - Traditional leader (society) - Group leader (women, youth etc.) - Member of the WASH committee - Other - No answer | | One choice | | |
| 10 | | Religious belief | | What is your religion? | |  | | - Christian - Muslim - Traditional believer - Other - No answer | | One choice | | |
| 11a | | Health status | | How would you describe your current physical well-being? | |  | | - Excellent - Good - Fair - Not good - Poor - No answer | | One choice | | |
| 11b | | Health status | | Have you suffered from diarrhoea in the past month? | |  | | - Yes - No - No answer | | One choice | | |
| 11c | | Health status | | Has anyone else in your family suffered from diarrhoea in the past month? | |  | | - Yes - No - No answer | | One choice | | |
| 12 | | Perceived barriers & constraints | | Please listen to the following statements and select for each statement how strongly you agree or disagree. | | - I don’t want my children to wash their hands because they waste water. (individual) - Soap is too expensive for everyday use of washing hands. (individual) - My family can’t afford a handwashing station. (individual) - The distance to the well is too far to fetch additional water for handwashing. (physical environment) - It takes too much time to wash my hands with soap each time I prepare food or touch food. (individual) - Sometimes I have more important things to do than washing hands. (individual) - I don’t always to remember to wash my hands. (individual) | | - Strongly disagree - Disagree - Undecided - Agree - Strongly agree - No answer | | Use visual scale; one choice per item | | |
| 13 | | Efficacy (self-efficacy and response efficacy) | | Please listen to the following statements and select for each statement how strongly you agree or disagree. | | - I am able to wash my hands in the critical moments of handwashing. (self-efficacy) - I am confident that I can perform proper handwashing. (self-efficacy) - I am certain I can remember handwashing after coming from the toilet or before touching food. (self-efficacy) - I can prevent diarrhoea when I wash my hands after going to the toilet. (response efficacy) - I believe I can do something to protect my own and other people’s health. (response efficacy) - I believe that washing my hands will keep me healthy. (response efficacy). | | - Strongly disagree - Disagree - Undecided - Agree - Strongly agree - No answer | | Use visual scale; one choice per item | | |
| 14 | | Feelings associated with handwashing (positive and negative) | | Please listen to the following statements and select for each statement how strongly you agree or disagree. | | - I don’t feel clean if I forget to wash my hands after going to the toilet. (disgust,) - Bad smell on my hands make me want to wash them with soap. (disgust) - Handwashing always reminds me of the Ebola epidemic. (fear) - I believe my family will be healthier if I wash my hands with soap all the time. (nurture) - I don’t feel good when I do not wash my hands with soap. (disgust) | | - Strongly disagree - Disagree - Undecided - Agree - Strongly agree - No answer | | Use visual scale; one choice per item | | |
| 15 | | Habit | | Please listen to the following statements and select for each statement how strongly you agree or disagree. | | - Once you take up the practice of hand-washing with soap, it’s easy to do it all the time. - Seeing soap after having been to toilet reminds me to wash my hands with soap. - Seeing dirt on my hands makes me wash them automatically. - Sometimes I miss out on hand hygiene simply because I forget it. - Handwashing is not a part of myself. | | - Strongly disagree - Disagree - Undecided - Agree - Strongly agree - No answer | | Use visual scale; one choice per item | | |
| 16 | | Social norms and support | | Please listen to the following statements and select for each statement how strongly you agree or disagree. | | - I would be ashamed if someone saw me not washing my hands after the toilet (social norms) - People will comment if I do not have clean hands and fingernails (social norms) - Visitors will respect me if they find a place to hand-wash in my home (social norms) - If I can´t afford to buy soap one day, my neighbour will help me. (social support) | | - Strongly disagree - Disagree - Undecided - Agree - Strongly agree - No answer | | Use visual scale; one choice per item | | |
| 17 | | Perceived consequences of diarrhea | | Please listen to the following statements and select for each statement how strongly you agree or disagree. | | - If I get sick from diarrhoea, I will feel weak. - Children will perform worse in school if they have diarrhoea. - My work performance will be affected, if I have diarrhoea. - My family and I will have higher expenditures for medical costs when someone has diarrhoea. | | - Strongly disagree - Disagree - Undecided - Agree - Strongly agree - No answer | | Use visual scale; one choice per item | | |
| 18a | | Knowledge | | In your view, what is the purpose of handwashing? | |  | | - Protects my health - Stops the spreading of diseases - Removes visible and invisible dirt/ contamination - Reduces the risk of diarrhea - Prevents children from getting sick - Allows for cleanliness & good body scent - Was only necessary during the Ebola outbreak - Is required for religious reasons - Other | | Open question, answers to be classified based on prompts, multiple choices, check each item that has been mentioned |  |  |
| 18b | | Knowledge | | What is the purpose of using soap when we wash our hands? | |  | | - After the toilet, there may be unseen contamination on my hands. Soap removes this invisible dirt (positive) - I need to use soap after using the toilet and before eating - There is no need for handwashing with soap. Water is sufficient. - Soap helps me to have a good body scent and clean body. - Other | | Open question, answers to be classified based on prompts, multiple choices, check each item that has been mentioned |  |  |
| 19 | | Perceived benefits | | What are some of the things that will happen to you and your family if your wash your hands frequently? | |  | | - to protect myself from germs and dirt - to prevent sicknesses such as diarrhea - to maintain health and happiness in life - to protect the health of those around me - to maintain cleanliness - to maintain spiritual cleanliness - to have a better future - to improve school performance - to work better - to spend lower costs for medical expenses | | Open question, answers to be classified based on prompts, multiple choices, check each item that has been mentioned |  |  |
| 20a | | Hand washing practice, station | | Do you have a place to wash your hands at your house? | |  | | - Yes - No - No answer | | One choice |  |  |
| 20b | | Hand washing practice, station | | If yes, what do you use? | |  | | - Veronica bucket - Tippy tap - Rubber/bucket without tap - Cooler - Other | |  |  |  |
| 20c | | Hand washing practice, frequency | | Let us think of yesterday. How often did you wash your hands yesterday? | |  | | - not at all - once or twice - 3-5 times - more than 5 times - No answer | | One choice |  |  |
| 21 | | Hand washing practice,  critical moments | | What made you wash your hands yesterday?  Follow-up: Can you think of any other situations when you should wash your hands? | |  | | - Before, during, and after preparing food - Before eating food - Before and after caring for someone who is sick - Before and after treating a cut or wound - Before breastfeeding - Before feeding a child - After using the toilet - After cleaning a child - After blowing your nose, coughing, or sneezing - After touching an animal, animal feed, or animal waste - After handling meat or fish - After touching garbage - After coming from the field - After eating - Before saying prayers | | Open question, answers to be classified based on prompts, multiple choices, check each item that has been mentioned |  |  |
| 22 | | Hand washing behaviour, steps | | Can you please describe the different steps of hand washing? | |  | | - Wet both hands - Apply soap and scrub - Rinse clean - Dry hands - Steps mentioned in right order - Some steps mentioned - False order | | Open question, answers to be classified based on prompts, multiple choices, check each item that has been mentioned |  |  |
